# Abnormality in Peripheral and Brain Iron Contents and the Relationship with Grey Matter Volumes in Major Depressive Disorder

**DOI:** 10.3390/nu16132073

**Published:** 2024-06-28

**Authors:** Wenjia Liang, Bo Zhou, Zhongyan Miao, Xi Liu, Shuwei Liu

**Affiliations:** 1 Shandong Key Laboratory of Mental Disorders, Department of Anatomy and Neurobiology, Institute for Sectional Anatomy and Digital Human, School of Basic Medical Sciences, Cheeloo College of Medicine, Shandong University, Jinan 250012, Shandong, China; 2 Institute of Brain and Brain-Inspired Science, Shandong University, Jinan 250012, Shandong, China; 3 Key Laboratory for Experimental Teratology of the Ministry of Education and Center for Experimental Nuclear Medicine, School of Basic Medical Sciences, Cheeloo College of Medicine, Shandong University, Jinan 250012, Shandong, China; 4 Department of Radiology, Shandong Mental Health Center, Shandong University, Jinan 250014, Shandong, China; 5 Department of Psychiatry, Shandong Mental Health Center, Shandong University, Jinan 250014, Shandong, China

**Keywords:** iron, QSM, major depressive disorder, plasma ferritin, GMV

## Abstract

Major depressive disorder (MDD) is a prevalent mental illness globally, yet its etiology remains largely elusive. Recent interest in the scientific community has focused on the correlation between the disruption of iron homeostasis and MDD. Prior studies have revealed anomalous levels of iron in both peripheral blood and the brain of MDD patients; however, these findings are not consistent. This study involved 95 MDD patients aged 18–35 and 66 sex- and age-matched healthy controls (HCs) who underwent 3D-T1 and quantitative susceptibility mapping (QSM) sequence scans to assess grey matter volume (GMV) and brain iron concentration, respectively. Plasma ferritin (pF) levels were measured in a subset of 49 MDD individuals and 41 HCs using the Enzyme-linked immunosorbent assay (ELISA), whose blood data were simultaneously collected. We hypothesize that morphological brain changes in MDD patients are related to abnormal regulation of iron levels in the brain and periphery. Multimodal canonical correlation analysis plus joint independent component analysis (MCCA+jICA) algorithm was mainly used to investigate the covariation patterns between the brain iron concentration and GMV. The results of “MCCA+jICA” showed that the QSM values in bilateral globus pallidus and caudate nucleus of MDD patients were lower than HCs. While in the bilateral thalamus and putamen, the QSM values in MDD patients were higher than in HCs. The GMV values of these brain regions showed a significant positive correlation with QSM. The GMV values of bilateral putamen were found to be increased in MDD patients compared with HCs. A small portion of the thalamus showed reduced GMV values in MDD patients compared to HCs. Furthermore, the region of interest (ROI)-based comparison results in the basal ganglia structures align with the outcomes obtained from the “MCCA+jICA” analysis. The ELISA results indicated that the levels of pF in MDD patients were higher than those in HCs. Correlation analysis revealed that the increase in pF was positively correlated with the iron content in the left thalamus. Finally, the covariation patterns obtained from “MCCA+jICA” analysis as classification features effectively differentiated MDD patients from HCs in the support vector machine (SVM) model. Our findings indicate that elevated peripheral ferritin in MDD patients may disrupt the normal metabolism of iron in the brain, leading to abnormal changes in brain iron levels and GMV.

## 1. Introduction

Major depressive disorder (MDD) is one of the most common mental disorders worldwide, characterized by enduring sadness and loss of interest. It is often accompanied by anxiety, physical discomfort and sleep disturbances, which seriously affect the daily functioning of people [1]. The onset of MDD is multifactorial, involving genetic, environmental, psychological and biological elements, with consensus yet to be reached on a definitive cause [2]. Recently, mounting evidence underscores the significance of dietary factors in MDD [3,4]. Iron, obtained through dietary intake, is a crucial trace element for the development and functionality of the brain. Abnormalities in the distribution and concentration of iron in the brain may contribute to the occurrence of psychiatric disorders, such as MDD [5,6,7]. Further research is warranted to explore the association between iron levels and MDD.

Iron plays multiple roles in the brain, including myelin sheath formation, neurotransmitter synthesis, oxygen transport, electron transfer, and more, which render this metal essential for life [8,9]. Even slight disturbances in iron balance can have a profound impact on organisms, particularly for brain, which is highly metabolically active and exceptionally sensitive to fluctuations in iron homeostasis [6]. Both iron deficiency and excess in the brain can impair motor and cognitive functions by affecting neurotransmitters such as serotonin, noradrenaline and dopamine, finally triggering the occurrence of various mental diseases [7,10,11]. Consistent with these preclinical findings, some observational and intervention studies have shown an association between abnormal iron levels and depressive symptoms. Zeng et al. gave mice subject to chronic unpredictable mild stress (CUMS) a high-iron diet and found that the elevated serum iron levels in mice were significantly correlated with impaired hippocampal functional connectivity (FC) and further induced the development of CUMS [12]. A study on children with Thalassemia (TDT) showed that iron overload induced by transfusion treatment may be causally related to major depression-like episodes (MDLEs) in this population [13]. Another study has reported elevated serum ferritin levels in some patients with depression, and significant improvement in depressive symptoms after treatment with iron chelators [14]. However, there are also some studies documenting the influence of iron deficiency on depression and depressive-like behaviors. Yi et al. found that middle-aged male workers in Japan with lower levels of serum ferritin concentration have a higher prevalence of depressive symptoms [15]. Another pilot study suggested that lower serum ferritin concentration potentially disrupted the iron supply in the brain, which may contribute to the emergence of MDD or anxiety disorder in adolescents [16]. Finally, a review implied a reduction in the severity of depression symptoms among women with postpartum depression when supplementing iron, especially among those suffering from iron deficiency [17]. Nevertheless, considering the interference factors in iron transport, uptakeandstorage, the concentration of peripheral ferritin may not accurately reflect the iron content in the brain [18]. While directly measuring brain iron levels through histological tissue analysis is a precise method, challenges in obtaining brain tissue have sparked interest in exploring non-invasive magnetic resonance imaging (MRI) techniques for accurate detection of brain iron content in vivo.

Quantitative susceptibility mapping (QSM) has emerged as a novel MRI technique to quantify the spatial distribution of magnetic susceptibility in biological tissues, which mainly utilizes the phase information of MRI to detect and quantitatively calculate the magnetic susceptibility values of magnetic-sensitive substances [19]. The magnetic susceptibility values correlate positively with the average iron levels in biological tissues and can identify tiny variations in iron levels of the brain in attention-deficit hyperactivity disorder (ADHD), autism spectrum disorder (ASD) and Parkinson’s disease (PD) [20,21,22]. Previous studies have also detected brain iron levels of MDD patients using QSM and observed elevated iron concentrations in certain brain regions of individuals with MDD [23,24,25]. However, these studies only have limited intergroup comparisons of QSM values in specific brain regions and have not explored the brain as a whole. Furthermore, no research has been conducted on the association of brain iron content with peripheral ferritin levels in the same group of subjects.

Therefore, the present study compares the iron concentration in the whole brain range of MDD patients and healthy controls (HCs) using QSM, especially the deep gray matter (DGM) nuclei, where iron depositions are more abundant in normal physiological conditions [5]. Additionally, Enzyme-linked immunosorbent assay (ELISA) kits were used to measure the plasma ferritin levels of each subject, and further correlation analysis was conducted between plasma ferritin (pF) levels and iron concentrations in DGM. Considering the established association between altered brain morphometry and MDD [26,27,28,29], and the impact of iron abnormality on neurotransmitter signaling within the brain [30,31], we also investigated the relationship between brain iron content and grey matter volume (GMV) using the multimodal canonical correlation analysis plus joint independent component analysis (MCCA+jICA) algorithm [32]. Finally, we input multidimensional features related to depression into a support vector machine (SVM) model to verify if it can effectively differentiate MDD from HCs.

## 2. Materials and Methods

### 2.1. Subjects

A total of 95 unmedicated patients with MDD (50 males, 45 females, aged 24.33 ± 4.63) and 66 HC participants (30 males, 36 females, aged 24.59 ± 2.637) were enrolled for MRI scans to measure brain iron and GMV. All participants underwent an unstructured interview with a certified psychiatrist. The detailed inclusion and exclusion criteria are outlined in Supplementary Table S1. All procedures were approved by the Ethics Committee of the School of Basic Medical Sciences at Shandong University (Reference Number ECSBMSSDU2022-1-61, 16 April 2022). Participation in the study was voluntary, and all subjects provided written informed consent in accordance with the Declaration of Helsinki.

### 2.2. MRI Experiments

All experiments were performed on a 3.0T MRI scanner (Discovery MR750, GE Healthcare, Milwaukee, WI, USA) with a 24-channel head coil. High-resolution T1-weighted images for anatomical reference were acquired using a three-dimensional BRAVO sequence with 192 slices (TR/TE, 8.488/3.248 ms; field of view (FOV), 256 × 256 mm^2^; matrix, 256 × 256; slice thickness, 1 mm). Three-dimensional spoiled gradient echo-based QSM imaging was performed with scan parameters as follows: number of TEs = 8 (first TE = 1.932 ms, TE interval = 4.6 ms), TR = 21.5 ms, FOV = 256 × 256 mm^2^, flip angle = 20°, matrix size = 256 × 256, slice thickness = 1 mm, number of slices = 140, scanning time = 4 min 38 s.

### 2.3. Calculation of QSM and GMV Metrics

At first, QSM maps were calculated from magnitude and phase data using STI Suite software, version 3.0 on the MATLAB 2018b (MathWorks, Natick, MA, USA) platform [33]. Subsequently, T1-weighted images were registered to the QSM space and then transformed to the MNI standard space using Advanced Normalization Tools (ANTs, http://stnava.github.io/ANTs/, accessed on 23 September 2023) software, version 2.5.0. Next, the QSM images were also registered to the MNI standard space utilizing the registration matrix of T1 maps. Finally, for the region of interest (ROI)-based analysis, mean susceptibility values of fourteen ROIs were obtained according to the Harvard–Oxford subcortical structural atlas (HOSubCort Atlas), including the bilateral amygdala, hippocampus (HIP), nucleus accumbens (NAcc), thalamus (THA), caudate nucleus (CN), putamen (PU), globus pallidus (GP), and used to make a representative comparison of concentration of iron in the brain between MDD and HC groups.

The GMV metric was obtained by segmenting, normalizing, and smoothing the structural MRI (sMRI) data using the CAT12 software, version 12.8.2 (https://neuro-jena.github.io/cat/, accessed on 28 June 2023). Specifically, T1-weighted data were segmented into grey and white matter as well as cerebrospinal fluid (CSF) using a unified segmentation approach [34] and spatially normalized into the MNI template. The intensity inhomogeneity of low-frequency images was also corrected using the N4 algorithm provided in the ANTs software. Then, the acquired images need to undergo smoothing with an 8 mm Gaussian kernel before statistical analysis. Finally, the GMV values of fourteen brain regions were also obtained through HOSubCort Atlas.

### 2.4. Plasma Ferritin Measurements

A total of 49 MDD and 41HCs voluntarily provided their blood samples during the MRI scanning session. Participants’ venous blood was collected in the morning following a minimum fast of 9 h, and subsequently centrifuged to isolate plasma. A sandwich method was used to determine the human pF levels through an ELISA reagent kit (KYY-0962H1, Keyybio, Jinan, Shandong, China). Purified human ferritin antibodies were coated on microplates to form solid-phase antibodies. Ferritin was sequentially added to the wells coated with monoclonal antibodies and then combined with Horseradish peroxidase (HRP)-labeled ferritin antibodies to obtain an antibody–antigen–enzyme–labeled antibody complex. After thorough washing, the substrate Tetramethylbenzidine (TMB) was added for color development. TMB turns blue in the presence of the HRP enzyme and is converted to the final yellow color under acidic conditions. The intensity of the color is positively correlated with the level of pF. The absorbance (OD value) was measured at a wavelength of 450 nm using a microplate reader, and the concentration of human pF was calculated using a standard curve.

### 2.5. The Fusion of QSM and GMV Metrics

In our study, QSM and T1 sequences were respectively utilized to explore the abnormal iron content and GMV in the brains of MDD patients. Considering the redundancy of high-dimensional MRI data, we used the “MCCA+jICA” method to extract features from these two modalities (QSM and GMV for sMRI) separately. These features are utilized for evaluating abnormalities in GMV and brain iron content, with features from diverse modalities displaying high correlation.

**Figure 1:**
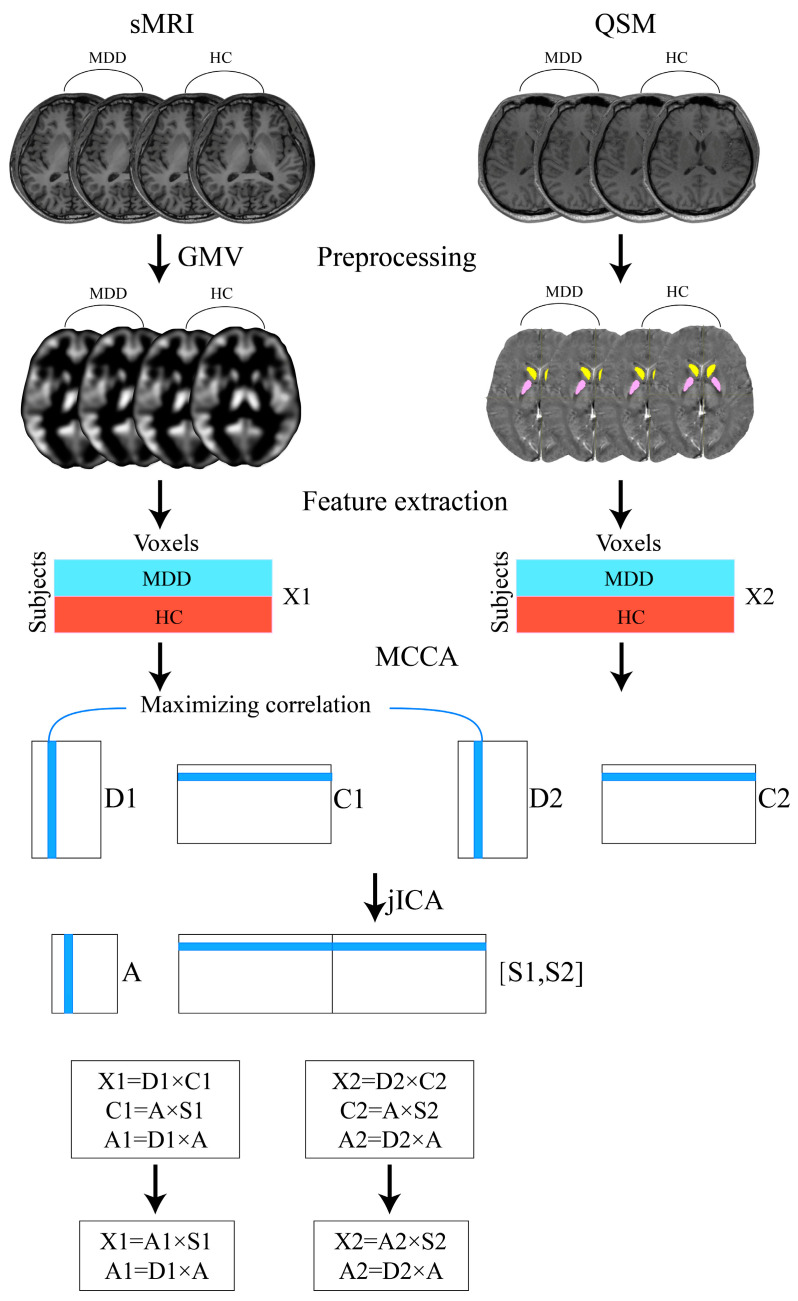
Pipeline of ‘MCCA+jICA’ for fusion analysis of structural MRI (sMRI) and quantitative susceptibility mapping (QSM) images.

“MCCA+jICA” is a fusion analysis model that maximizes the correlation between different MRI features and examines the between-group differences; this was performed using the Fusion ICA Toolbox (FITv2.0e, http://mialab.mrn.org/software/fit/, accessed on 12 September 2023). As shown in Figure 1, the specific steps were as follows: after feature extraction, sMRI and QSM modal features were rearranged into feature matrices X1 and X2, respectively, with dimensions of N × M. Here, N represents the number of subjects and M denotes the number of voxels. Subsequently, the two feature matrices were standardized into Z-scores to minimize the range disparities between sMRI and QSM metrics [35]. The number of independent components (ICs) was estimated for each MRI feature according to an improved minimum description length (MDL) criterion after normalization [36]. Singular value decomposition (SVD) was then used to reduce the dimensionality of the feature matrices. Next, MCCA was utilized to derive canonical variant matrices (D1, D2) from the dimensionally reduced matrices, along with the associated components matrices (C1, C2) for sMRI and QSM, respectively. The jICA algorithm was performed on the C1 and C2 matrices to obtain the mixing coefficient matrix A and maximized joint ICs (S1, S2). The product of the canonical variant matrix and mixing coefficient matrix is the final mixing coefficient matrix (sMRI: A1 = D1 × A; QSM: A2 = D2 × A). Each column of the final mixing coefficient matrices (A1 and A2) was subjected to a two-sample *t*-test to compare the group differences between MDD patients and HCs. Those ICs that exhibit significant group differences in the mixing coefficients between MDD and HC groups are called group-discriminative ICs. Finally, we aimed to find a joint group-discriminative IC that can differentiate MDD and HC groups with statistical significance in both sMRI and QSM modalities [35,37]. False discovery rate (FDR) correction was applied for multiple comparisons.

### 2.6. SVM for Classification

Based on the results of the “MCCA+jICA” analysis, the QSM and GMV components were classified into positive (Z > 0) and negative (Z < 0) feature masks, which were subsequently clustered. Multiple clusters were derived for each modality and employed as ROIs for feature extraction from individual participants. Mean values of the multimodal indicators within each ROI, in conjunction with the age and sex information for each participant, were calculated, resulting in a feature matrix with dimensions N_subj_ × M_(roi+2)_. Here, 95 MDD patients and 66 HCs were recruited for MRI scanning. Therefore, the feature matrix dimension is 161 × 28. Then, the 161 × 28 brain features were input into the SVM model to predict the diagnostic status of participants, categorizing them as either individuals with MDD or HCs. Here, 49 MDD and 41 HC subjects were used as the training set with both MRI and blood data, while the remaining 46 MDD and 25 HC subjects were set aside as an independent test set. For individual-level classification, a structural feature abnormalities (SFA) score was defined to quantify the deviation of an individual’s structural features from those of the HC group. This index was determined by measuring each individual’s distance from the separating hyperplane based on hyperparameters that yielded optimal classification outcomes. When the SFA score is above 0, the individual is classified as MDD. Conversely, if the score is below 0, the individual is considered HC. Finally, the SFA score was, as the test variable, input into the receiver operating characteristic (ROC) curve to further show the classification results between MDD and HC groups in the test set. The concept for proposing this novel metric was primarily influenced by the prior research conducted by Li et al. [38].

### 2.7. Statistical Analysis

SPSS software, version 22.0 (IBM, Armonk, NY, USA) and GraphPad Prism 9.0 were used for statistical analysis. In our analysis, we tested the normality of distributions using the Shapiro–Wilk test before further statistical detection and used standard parametric statistics to draw statistical inferences only when a Gaussian distribution was confirmed for each dependent variable.

The chi-square test was used to compare the sex and relevant clinical information, such as Self-injury behavior (yes/no), Suicidal thoughts (yes/no), Suicidal behavior (yes/no) and Childhood trauma (yes/no), between the MDD and HC groups. The independent samples *t*-test was used to compare age, body mass index (BMI), Hamilton Rating Scale for Depression (HAMD), Beck Depression Inventory (BDI), Hamilton Rating Scale for Anxiety (HAMA) and brain iron content and GMV values of the ROIs in MDD patients and HC subjects. All participants who contributed plasma samples underwent measurement of their pF levels, and analysis of covariance (ANCOVA) was employed to examine the disparities in pF levels between the MDD and the HC groups (age and sex as the covariates). Pearson’s correlation analysis was separately performed to assess the relationships of susceptibility values with GMV and pF. The relationship between SFA metric and clinical information, like HAMD and BDI, was also examined using Pearson’s correlation analysis. The Significant threshold was set at *p* < 0.05.

## 3. Results

### 3.1. Demographic and Clinical Characteristics

Table 1 shows the detailed demographic and clinical information of 95 MDD patients and 66 HCs, all of whom underwent T1 and QSM MR sequence scans. Among them, 49 MDD patients and 41 HCs underwent concurrent venous blood collection during the MR scans. There were no notable differences in fundamental characteristics such as age, sex, and BMI. However, significant intergroup differences were observed in clinical rating scales, including HAMD, HAMA, BDI and other assessments.

### 3.2. The Fusion of QSM and GMV Metrics

**Figure 2:**
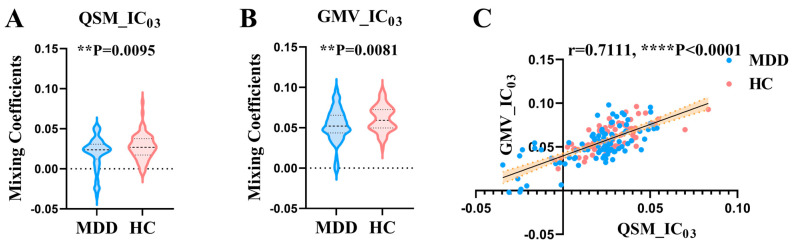
The jointdiscriminative components IC_03_ for QSM and GMV metrics were identified using the “MCCA+jICA” algorithm. (**A**,**B**) The mean mixing coefficients of MDD participants were adjusted lower than HC subjects for QSM and GMV metrics. (**C**) The QSM_IC_03_ was significantly positively correlated with GMV_IC_03_. IC = independent component; QSM = quantitative susceptibility mapping; GMV = grey matter volume; MCCA+jICA = Multimodal canonical correlation analysis plus joint independent component analysis; MDD = major depressive disorder; HC = healthy control. The displayed *p* values passed the FDR correction for multiple comparisons.

**Figure 3:**
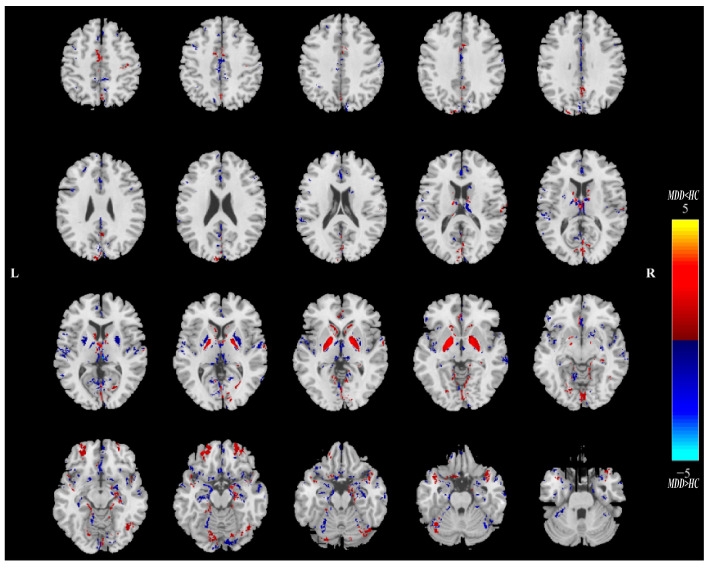
The spatial maps of QSM_IC_03_ visualized at |Z| > 2, where the red brain regions (Z > 0) indicate higher QSM values in HC subjects than MDD participants, and the blue brain regions (Z < 0) indicate higher QSM values in MDD subjects than HC participants. MDD = major depressive disorder; HC = healthy control.

**Figure 4:**
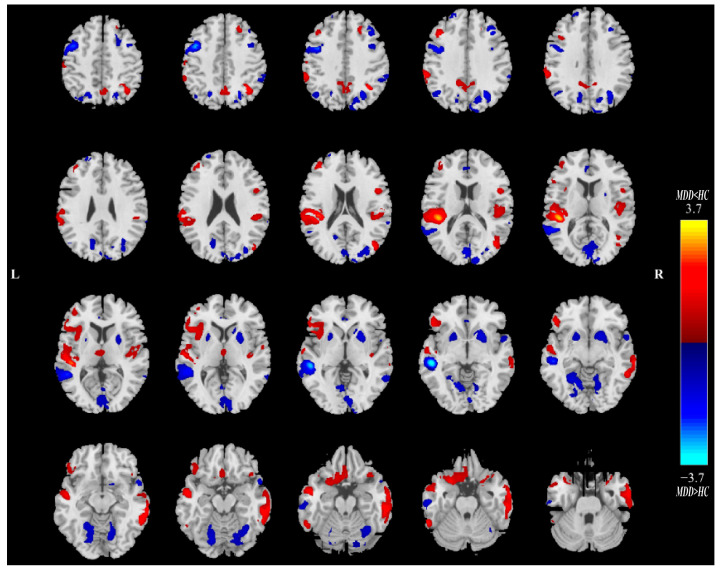
The spatial maps for GMV_IC_03_ visualized at |Z| > 2, where the red brain regions (Z > 0) mean higher GMV values in HC subjects than MDD participants, and the blue brain regions (Z < 0) indicate higher GMV values in MDD subjects than HC participants.; MDD = major depressive disorder; HC = healthy control.

Twenty ICs for QSM and sMRI were estimated in this study. Intergroup statistics were applied using the two-sample *t*-test to analyze the mixing coefficients of each IC. As shown in Figure 2, the third independent component (IC_03_) can differentiate between the MDD and HC groups in terms of QSM and GMV. Meanwhile, there is a significant correlation between QSM_IC_03_ and GMV_IC_03_. Therefore, IC_03_ is the joint group-discriminative IC that we finally obtained. IC_03_ exhibited significant inter-group differences in QSM (** *p* = 0.0095) and GMV (** *p* = 0.0081) metrics, respectively (see Figure 2A,B). QSM_IC_03_ exhibited positive correlations with GMV_IC_03_ (r = 0.7111, **** *p* < 0.0001) (Figure 2C). These *p* values passed the FDR correction for multiple comparisons. Spatial maps of QSM_IC_03_ and GMV_IC_03_ were transformed into Z scores and are visualized at |Z| > 2 in Figure 3 and Figure 4. The mean mixing coefficients were adjusted as HC subjects > MDD participants for QSM and GMV (Figure 2A,B), meaning the red brain regions (Z > 0) represent areas where QSM and GMV values of the HC group were higher than the MDD group. In contrast, blue brain regions (Z < 0) indicate areas where QSM and GMV values of the MDD group were higher than the HC group (Figure 3 and Figure 4).

Overall, lower QSM values were observed in the bilateral CN, GP, middle frontal gyrus and other regions of MDD patients in the spatial map of QSM_IC_03_. For bilateral PU, THA, anterior cingulate cortex, superior temporal gyrus and parahippocampal gyrus, etc., the QSM values of MDD in these regions are greater than those of HCs (Figure 3). As for the GMV_IC_03_ (Figure 4), the GMV values of anterior cingulate cortex, bilateral PU, supramarginal gyrus and lingual gyrus in MDD patients increased compared with the HCs. In the right insula, bilateral superior temporal gyrus, right gyrus rectus, orbital gyrus and THA, the GMV values of MDD patients were lower than those of HCs.

### 3.3. The SVM Model Distinguished MDD Patients from HCs Well

The feature matrix composed of GMV and QSM values produced a good classification in MDD patients and HCs. Then the SFA score was obtained by calculating the distance of each individual to the separating hyperplane based on the optimal classification results. The individual was classified as MDD when the SFA score exceeded 0. Conversely, if the score was below 0, the individual was deemed to be a HC. Supplementary Figure S1 reveals that the majority of individuals were accurately classified. Additionally, the SFA score showed a significant positive correlation with HAMD. Furthermore, an ROC curve was plotted to illustrate the classification results between MDD and HC groups in the test set. Optimal diagnostic accuracy of 64.79% was achieved, with an Area Under the Curve (AUC) of 0.63.

### 3.4. Comparison Results of pF and ROI Analysis of Brain Iron Levels and GMV Values

**Figure 5:**
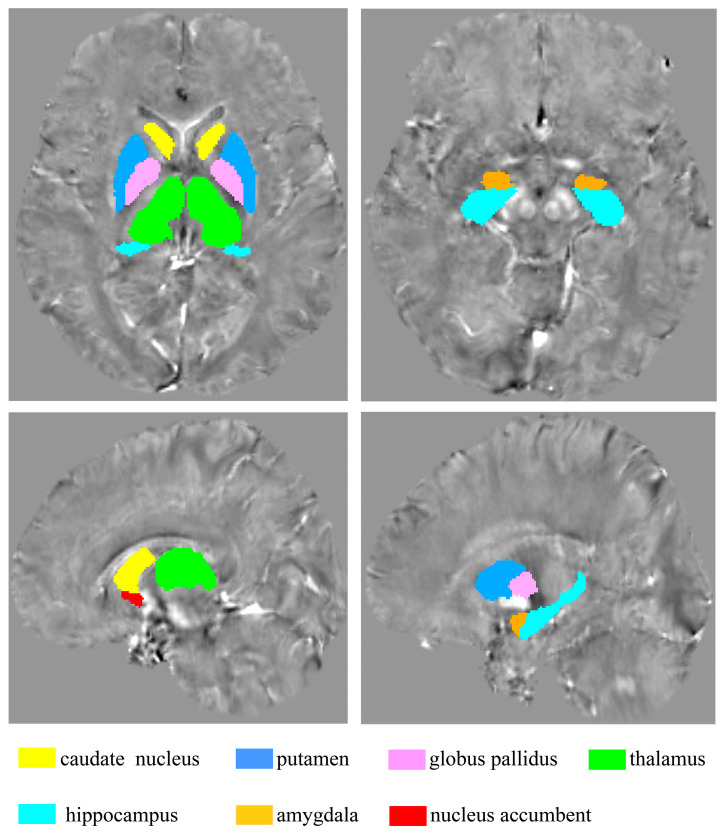
Fourteen ROIs were obtained according to the HOSubCort Atlas.

The ROI-based comparison of iron concentration in the brain between MDD and HC groups was conducted using fourteen ROIs delineated by the HOSubCort Atlas, as illustrated in Figure 5. This approach served to validate the results of the “MCCA+jICA” analysis.

**Figure 6:**
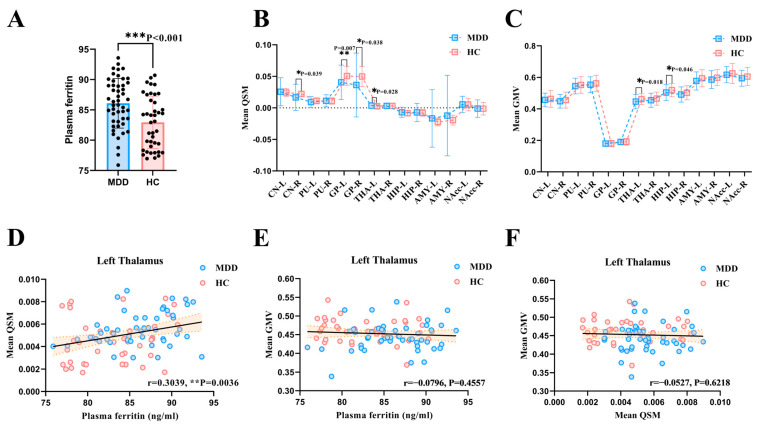
The comparison results of pF and ROI analysis of QSM and GMV values. (**A**) The pF levels in MDD patients were higher than those of HCs. (**B**) The mean QSM values in the right CN and bilateral GP of MDD patients were lower than those of HCs. However, the mean QSM values in the left THA of MDD patients were higher than those of HCs. (**C**) The mean GMV values in the left THA and left HIP of MDD patients were lower than HCs. (**D**) The pF contents were positively correlated with the mean QSM values of left THA. (**E**,**F**) No relationships were observed between GMV values of left THA and the pF contents. Similarly, no significant correlation was found between the average QSM value and the average GMV value of the left THA. QSM = quantitative susceptibility mapping; GMV = grey matter volume; MDD = major depressive disorder; HC = healthy control; CN = caudate nucleus; PU = putamen; GP = globus pallidus; THA = thalamus; HIP = hippocampus; AMY = amygdala; NAcc = nucleus accumbens.

The ELISA results showed that MDD participants had increased pF content compared with HCs (Figure 6A) (*** *p* < 0.001). The mean and standard deviation of pF in the MDD and HC groups are 86.09 ± 4.10 ng/mL and 82.95 ± 4.30 ng/mL, respectively. Statistical analysis revealed that the QSM values in the right CN (* *p* = 0.039), left GP (** *p* = 0.007) and right GP (* *p* = 0.038) of patients with MDD were significantly lower than those in the HC group. On the contrary, in the left THA (* *p* = 0.028), the QSM values of MDD patients were higher than those of HCs (Figure 6B). The GMV values in the left THA (* *p* = 0.018) and left HIP (* *p* = 0.046) of MDD patients were lower than HCs (Figure 6C). Pearson’s correlation analysis showed that the pF contents were significantly positively correlated with the mean QSM values of left THA (r = 0.3039, ** *p* = 0.0036) (Figure 6D). However, in the left THA, no relationships were observed between GMV values and the pF contents (Figure 6E). Similarly, no significant correlation was found between the average QSM value and the average GMV value of the left THA (Figure 6F).

## 4. Discussion

To our knowledge, this study is the first to investigate the association between brain iron concentration, plasma ferritin and brain structure in unmedicated MDD patients. The brain iron content and GMV of MDD patients were examined using the QSM and 3D-T1 sequences, respectively. At the same time, we used the ELISA kits to detect the concentration of pF. Our results showed that the ferritin concentrations in the peripheral plasma of individuals with MDD are significantly higher than those of HCs. The associations between GMV and QSM values in the brain were identified using the “MCCA+jICA” algorithm. Finally, GMV and QSM feature matrices were input into an SVM model for effective classification of MDD and HCs.

The “MCCA+jICA” algorithm is a data-driven method used for multivariate fusion, enabling the examination of interactions between various imaging modalities [35]. In this study, QSM_IC_03_ and GMV_IC_03_ were joint-discriminative components for MDD and HC groups, which suggest that abnormalities of brain iron content related to GMV are mainly concentrated in the CN, GP, PU, THA, parahippocampal gyrus and other basal ganglia regions. Regional iron overload can continuously generate toxic free radicals, damage dopamine synthesis and be harmful to brain motor and cognitive functions, thereby leading to the occurrence of various neurodegenerative disorders [39]. Previous studies found that brain iron is particularly concentrated in the basal ganglia regions, which is an area highly influenced by dopamine and gamma-aminobutyric acid (GABA) metabolism [40,41]. Therefore, the function of basal ganglia regions is very sensitive to changes in iron status. In recent years, the link between mental health disorders such as MDD and iron homeostasis in the basal ganglia regions has been preliminarily explored. Yao et al. manually drew the ROIs of basal ganglia and other DGM nuclei to compare the iron concentration of these regions between MDD patients and HCs. They found that MDD patients have significantly higher QSM values in the bilateral PU and left THA compared to HCs [24]. The same method was used by Zhang et al. to explore the iron content in the basal ganglia of elderly patients with MDD [25]. They found that the THA QSM values of MDD were significantly higher than those of HCs, suggesting a correlation between iron deposition in the THA and depressive symptoms in the elderly. Both of these studies are based on regional QSM studies of ROI. There are two flaws: firstly, the ROI definition is usually inaccurate due to the weak contrast of the QSM images and unclear anatomical boundaries. Secondly, the subjective ROI delineation and relatively poor measurement consistency may cause a certain degree of research bias. Duan et al. combined the results of whole-brain and regional QSM studies and found that in patients with recurrent depression, increased brain iron deposition occurred not only in two iron-rich areas, such as the PU and THA, but also in areas with minimal iron content (frontal lobe, temporal lobe, occipital lobe, hippocampus and cerebellum) [23]. The study suggests that the abnormal distribution pattern of brain iron deposition in MDD patients should draw our attention, even though its underlying mechanisms are not yet clear, and strongly indicates that research on brain iron should not focus solely on the DGM. Therefore, whole-brain and regional QSM analyses were both conducted in our study. The consistent results showed that the QSM values of left THA in unmedicated MDD patients were higher than HCs. Furthermore, our ELISA results showed a significantly higher concentration of pF in MDD patients compared to HCs. Additionally, this elevated pF concentration was significantly and positively correlated with the mean QSM values of the left thalamus. On the other hand, consistent results from both a whole-brain and regional GMV analysis showed the GMV of left THA in MDD patients was lower than in HCs. These above results all suggest a high correlation between abnormal iron content and GMV in the thalamus and the occurrence of mental disorders, especially MDD.

We also found iron deficiency in the bilateral CN, GP, middle frontal gyrus and other brain areas from our “MCCA+jICA” results. Iron is an essential trace element in the human body and plays a key role in the synthesis of various neurotransmitters, such as dopamine, serotonin, norepinephrine, glutamate and GABA [42]. Therefore, iron deficiency is closely associated with altered neurotransmission in the brain, especially with changes in monoamine metabolism [39]. Animal experiments have shown a strong correlation between abnormal behaviors resulting from iron deficiency in rodents and changes in dopamine metabolism in the striatum [43,44]. Iron deficiency may also affect the glutamatergic system and energy metabolism in early development [45,46]. Furthermore, many human studies have found that iron deficiency has a significant impact on human behavior and mental health during brain development [39]. Lozoff et al. found that children with iron deficiency exhibit increased anxiety and/or depressive symptoms, along with social and attentional issues [47]. During adolescence, iron deficiency may disrupt brain maturation and lead to the emergence of internalizing disorders such as anxiety or depression [16]. In addition, pregnant women with iron deficiency are at higher risk of postpartum depression, while iron supplementation can reduce the risk of depression [17].

The comparison results of GMV in the “MCCA+jICA” analysis showed that the volumes of the GMV values of anterior cingulate cortex, bilateral PU, supramarginal gyrus and lingual gyrus in MDD patients were increased. Previous studies demonstrated that iron deficiency can affect the normal structure and function of the brain. Abbas et al. found a negative correlation between body iron levels and the volumes of the PU and the left CN [16], which may be related to dopaminergic signaling damage triggered by iron deficiency [48]. Preclinical studies have found that an increase in PU volume may be associated with stimulant-induced reduction in dopamine D_2_ receptor density of the ventral striatum [49]. Moreover, Ersche and his colleagues found that patients with stimulant use disorders have an enlarged PU volume in the clinical practice [49]. Therefore, iron, as a cofactor for dopamine synthesis and storage, its abnormal changes have a high correlation with morphological changes in the brain, such as changes in GMV. However, further research is needed to confirm whether iron supplementation can reverse the changes in brain structure, in order to validate the causal relationship between iron and brain structural changes.

There are several limitations in this study. Firstly, the MRI results were obtained from a single-center study with a restricted sample size, and robust results require a larger amount of data for validation. Secondly, we should collect longitudinal data to further confirm the causal relationship between brain iron and GMV. Finally, we did not collect relevant diet information of MDD patients and HCs, which may affect the pF levels. We can collaborate with psychologists to make a professional diet scale in the future and re-investigate these subjects in our study, so as to better explain our results.

## 5. Conclusions

Overall, our research explores the diagnostic characteristics of patients with MDD in terms of plasma ferritin, brain iron content and GMV. We mainly analyze the correlation between brain iron content and GMV using the “MCCA+jICA” method. The alterations in iron content and GMV in the basal ganglia area of MDD patients were found to be notably pronounced. Subsequently, we input the relevant features into an SVM model for the classification of MDD and HCs. We also utilized ROI-based analysis for further validation. Our findings support the correlation between abnormal iron content and brain structural abnormalities in MDD patients, indicating that iron may serve as a potential biomarker for understanding the pathophysiological mechanisms of MDD.

## Figures and Tables

**Table 1:** Demographic information and clinical characteristics of participants included in this study.

	MDD (*n* = 95) Mean ± SD	HC (*n* = 66) Mean ± SD	t/χ^2^	*p*
Age (years)	24.33 ± 4.630	24.59 ± 2.637	0.4192	0.6756
Sex (male/female)	50/45	30/36	0.8024	0.3704
^a^ BMI (kg/m^2^)	22.06 ± 4.114	21.83 ± 3.151	0.3739	0.7090
^b^ HAMD-17	25.68 ± 5.547	3.409 ± 3.930	28.08	**** <0.0001
^c^ BDI-II	34.19 ± 10.82	4.864 ± 5.891	20.04	**** <0.0001
^d^ HAMA	27.81 ± 8.458	3.242 ± 4.413	21.63	**** <0.0001
Self-injury behavior (yes/no)	36/59	1/65	27.10	**** <0.0001
Suicidal thoughts (yes/no)	80/15	8/58	81.66	**** <0.0001
Suicidal behavior (yes/no)	22/73	0/66	15.79	**** <0.0001
Childhood trauma (yes/no)	20/75	2/64	9.249	** 0.0024

^a^ BMI: Body mass index, which was measured as weight (kg) divided by height squared (m^2^). ^b^ HAMD-17: Hamilton Rating Scale for Depression, a 17-item clinician-administered rating scale with a total score range of 0–52. ^c^ BDI-II: Beck Depression Inventory version 2, a 21-item self-report questionnaire, with a total score range of 0–63. ^d^ HAMA: Hamilton Rating Scale for Anxiety, a 14-item clinician-administered rating scale with a total score range of 0–56. **** *p* < 0.0001, ** *p* < 0.01.

## Data Availability

The data and code generated or analyzed during the study are available from the corresponding author, upon reasonable request.

## Supplementary Materials

The following supporting information can be downloaded at: https://www.mdpi.com/article/10.3390/nu16132073/s1, Figure S1: The diagnostic capability of the multimodal MRI patterns (QSM and GMV features) was evaluated; Table S1: The detailed inclusion and exclusion criteria for all subjects.
