# Supplementary material for: Abnormality in Peripheral and Brain Iron Contents and the Relationship with Grey Matter Volumes in Major Depressive Disorder
Source: Nutrients. 2024 Jun 28;16(13):2073. doi: 10.3390/nu16132073 (PMC11243628; doi:10.3390/nu16132073)
Supplement: Supplementary file 1 [file nutrients-16-02073-s001.zip › nutrients-3033704-supplementary.pdf]

Supplementary Table S1. The detailed inclusion and exclusion criteria for all subjects

Group	Inclusion Criteria	Exclusion Criteria
MDD	(1) The diagnosis of MDD should be made by middle-level doctors with psychiatric licenses according to the screening criteria of ICD-10;<br>(2) Onset of the disease for the first time and no previous psychiatric drug treatment;<br>(3) Hamilton Rating Scale for Depression (17 items) score >17;<br>(4) Aged 18 to 35 years old, understood and voluntarily signed a written informed consent.	(1) History of bipolar disorder or other non-affective psychotic disorders;<br>(2) Family history of mental illness, or history of substance abuse or alcoholism;<br>(3) History of cerebrovascular and other brain diseases, such as cerebral hemorrhage, cerebral infarction, epilepsy and brain tumors, etc.;<br>(4) Severely impaired vision/hearing or limb movement, which makes it impossible to complete the experiment;<br>(5) Contraindications to MRI, such as implanted cardiac pacemaker, metal denture, etc. Or the researchers deemed inappropriate for participants.
HC	(1) Aged 18 to 35 years old, understood and voluntarily signed a written informed consent;<br>(2) Hamilton Rating Scale for Depression (17 items) score < 7.	(1) History of major psychiatric disorders as defined by ICD-10;<br>(2) Family history of mental illness, or history of substance abuse or alcoholism;<br>(3) Contraindications to MRI, such as implanted cardiac pacemaker, metal denture, etc. Or the researchers deemed inappropriate for participants.

ICD-10: the tenth revision of the International Statistical Classification of Diseases and Related Health Problems.

Testing set

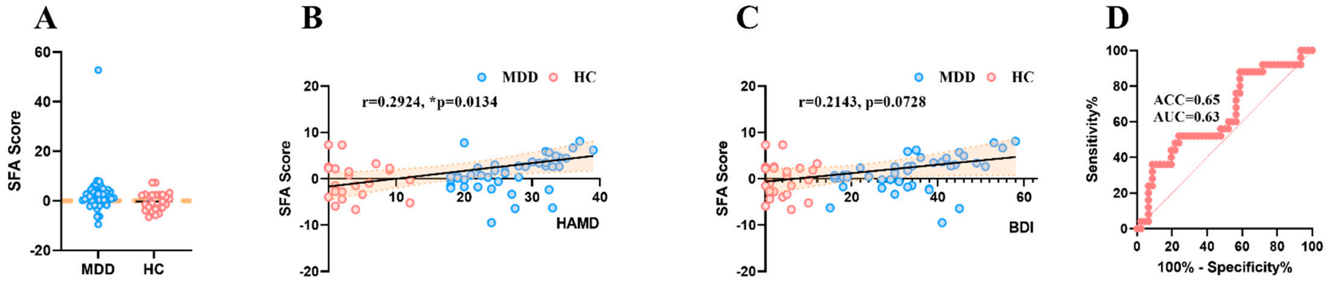

Supplementary Figure S1. The diagnostic capability of the multimodal MRI patterns (QSM and GMV features) was evaluated. **(A)** When SFA score exceeded 0, the individual was categorized as an MDD patient. Conversely, a subject was designated as a HC when the SFA score was below 0. Analysis of the bar chart demonstrates that a majority of individuals were accurately classified. **(B)** This SFA score exhibited a significant positive correlation with HAMD. **(C)** No significant correlation was found between the SFA score and BDI. **(D)** From the receiver operating characteristic (ROC) curve, we can see that the classification accuracy for the MDD and HC groups in the testing dataset is 65%, with an area under the curve of 0.63. SFA = structural feature abnormalities; MDD = major depressive disorder; HC = healthy control; HAMD = Hamilton Rating Scale for Depression; BDI = Beck Depression Inventory; ACC = accuracy; AUC = area under the curve.
